# Assessment of Women’s Satisfaction During Childbirth in a Tertiary Care Hospital in Central India

**DOI:** 10.7759/cureus.91214

**Published:** 2025-08-28

**Authors:** Smita Batni, Rashmi Bajpai, Aditi Singh, Neha Singh, Pooja Patil, Mitali Mishra, Pooja Kriplani

**Affiliations:** 1 Obstetrics and Gynaecology, Laxmi Narain (LN) Medical College, Bhopal, IND; 2 Anatomy, Laxmi Narain (LN) Medical College, Bhopal, IND; 3 Biochemistry, Laxmi Narain (LN) Medical College, Bhopal, IND

**Keywords:** attitude of health personnel, degree of freedom (df), early initiation of breastfeeding, maternal birth experience, maternal perception

## Abstract

Introduction

The main objective was to evaluate how satisfied women were with the care they received during labor, while the secondary aim focused on pinpointing specific aspects of labor care that required enhancement to create a more positive delivery experience.

Methodology

This observational research took place in the maternity ward of the Obstetrics and Gynecology department at L.N. Medical College and J.K. Hospital and Research Centre in Bhopal. During a six-month period from August 2024 to January 2025, 250 women participated in the research, which followed the successful completion of a preliminary pilot study involving 25 women (representing 10% of the total sample). The study included all low-risk expectant mothers carrying single babies in cephalic presentation at full term who gave birth either through vaginal delivery or cesarean section (both planned and emergency procedures) after providing proper consent. Women who delivered at home or while traveling to the hospital were not included. Data collection used a pre-designed format with a structured, previously validated questionnaire covering two main areas: demographic information and satisfaction evaluation, which was analyzed using the Likert score. The questionnaire contained 25 items organized into four main sections: health care (seven items); health worker communication (six items); health worker's attitude (five items); and environment (seven items). The statistical analysis was conducted using SPSS software version 29.

Results

The participant characteristics showed mostly younger women (average age 27.04±4.46 years), primarily 169 (67.6%) urban women, with diverse educational levels, with a majority of Hindi-speaking women (242, 96.0%), and 209 (83.6%) pregnancies were planned. In the study, 232 (92.8%) women expressed high satisfaction levels regarding the medical services provided. Although satisfaction remained elevated in the communication category, it demonstrated somewhat lower rates in 211 (84.4%) women when compared to the health care domain. Women showed high satisfaction with the healthcare worker's attitude (223, 89.2%), though how staff interacted with family members or companions and the degree of freedom allowed in the delivery ward received the poorest ratings in this category (220, 88.0%). The facility environment category demonstrated notably lower satisfaction compared to all other areas. Starting breastfeeding at the delivery location received the poorest satisfaction rating, with 138 (55.2%) women showing dissatisfaction.

Conclusion

Our research revealed that while medical care standards were adequately maintained, and there exists a clear requirement for improving respectful treatment toward both families and women overall. Quality healthcare combines effective medical treatment with proper staff communication, positive attitudes, and adequate institutional support.

## Introduction

The complex dimensions of healthcare delivery quality and patient satisfaction serve as crucial measures for evaluating care standards [1,2]. For women, their contentment with current birthing and delivery services becomes the strongest factor influencing their selection of medical facilities, adherence to recommended treatments, participation in follow-up appointments, and prompt identification plus management of complications throughout pregnancy, labor, and postpartum phases [3-7]. Enhancing maternal healthcare services within a nation's medical system holds significant importance in decreasing deaths and health issues among mothers that stem from pregnancy-related complications, labor difficulties, and delivery challenges.

When women view their childbirth and delivery care favorably, this connects to beneficial outcomes for both mother and baby's mental and physical well-being. These positive results include improved breastfeeding success, stronger maternal-infant connections, and reduced likelihood of future pregnancy terminations [8]. Conversely, when women evaluate their care negatively, this links to adverse consequences, including postpartum psychological challenges (such as postnatal depression and post-traumatic stress disorder), preference for surgical delivery, and difficulties with breastfeeding [8].

Enhancing care quality also plays a vital role in reaching Universal Health Coverage goals by 2030 [9]. The transformative healthcare approach outlined in the Global Strategy for Women's, Children's and Adolescent Health demands that maternity care extend beyond simply ensuring survival during delivery [10]. Recognizing which results matter most to women is essential for creating clinical standards and policies that prioritize women's needs, increasing the likelihood that mothers, infants, and families will flourish rather than merely survive after birth. The goal is achieving meaningful transformation in their lives and those of their families and communities, both immediately and over time [11]. Consequently, employing proven and dependable tools to evaluate women's birthing experiences is crucial for ensuring and fostering respectful and compassionate care [12]. The main aim of this research was to evaluate how satisfied women were with the care they received during labor, while the secondary aim focused on pinpointing specific aspects of labor care that require improvement to enhance the overall birthing experience.

## Materials and methods

This observational cross-sectional study was conducted in the labor room ward of the Department of Obstetrics and Gynecology at LN Medical College and JK Hospital and Research Centre, Bhopal, India, after obtaining approval from the Institutional Ethics Committee of LN Medical College and JK Hospital (Reference Letter No. LNMC&RC/Dean/2024/Ethics/250). Over the period of six months from August 2024 to January 2025, 250 women were enrolled in the study after a pilot study was completed with 25 women (10% of the sample size) successfully. All low-risk antenatal women with singleton cephalic presentations at term were included in the study, irrespective of their route of delivery, that is, by cesarean section (elective and emergency) or by vaginal route, after giving due consent. All those who delivered at home or in transit to the hospital were excluded. The primary objective of this study was to assess women's satisfaction with the care they received during labor, while the secondary objective was to identify particular areas of labor care that need improvement to enhance the overall childbirth experience.

All information was collected using a pre-designed tool featuring a structured questionnaire that covered two main areas: (i) socio-demographic details and (ii) satisfaction evaluation. The survey instrument was a structured tool, which was internally validated at the institutional level by a group of experts for consistency (reliability and validity). This was adapted for the Indian context and was available in both English and Hindi (local language). Researchers personally administered this questionnaire face-to-face to women during the initial 24 hours following delivery while they were still in the labor ward. Each completed form received numerical coding to ensure participant privacy remained protected.

The study tool consisted of 25 items/variables grouped into four categories: health care (seven questions); health worker communication (six questions); health worker's attitude (five questions); and environment (seven questions). Each survey question was evaluated using a five-point Likert scale score (one=completely dissatisfied; two=dissatisfied; three=neutral; four= satisfied and five=completely satisfied). The resulting scores obtained were dichotomized: scores of four and five being merged as ‘satisfied’ and those of one to three being categorized as ‘unsatisfied’. Neutral responses were classified as dissatisfied, considering that they may represent an ambiguous way of expressing dissatisfaction.

For an individual woman, a domain’s overall satisfaction was termed either ‘high satisfaction’ or ‘low satisfaction’. The total number of questions in every domain was considered as 100% satisfaction with each response termed as satisfied, given a score of one and unsatisfied given a score of zero. For the overall domain, it was taken that any total score of a domain less than 75% was considered to show low satisfaction. All four domains were studied independently for each woman.

Hence, for example, if there were seven questions in the domain, then the total score seven for the overall domain for a woman means the highest satisfaction (100%). Any total score of more than 5.25 (75% of seven) will be high satisfaction and any total score of 5.25 or less (75% of seven) will be low satisfaction for that woman in that domain.

Statistical analysis was conducted using SPSS software version 29 (IBM Corp, Armonk, NY). Information in categories was shown through frequencies and percentages. Numerical data was displayed as means alongside standard deviations. 

## Results

The study population consisted of predominantly young women (mean age 27.04±4.46 years), 169 (67.6%) urban women, with a wide range of educational backgrounds, and primarily Hindi-speaking women (242, 96.0%). Most of the women had given birth before and were unemployed. The large majority of the pregnancies were planned (209, 83.6%) (Table 1).

**Table 1:** Distribution of women according to demographic variables

S. No.	Demographic variables	N (%)
1	Age	
	18-25 years	113 (45.2)
	26-35 years	127 (50.8)
	>35 years	10 (4.0)
2	Educational status	
	Illiterate	5 (2.0)
	Up to senior secondary	124 (49.6)
	Graduate	82 (32.8)
	Postgraduate	39 (15.6)
3	Rural/urban	
	Rural	81 (32.4)
	Urban	169 (67.6)
4	Parity	
	Para 1	110 (44.0)
	Para 2	89 (35.6)
	Para 3	36 (14.4)
	Para >3	15 (6.0)
5	Language of preference	
	English	8 (4.0)
	Hindi	242 (96.0)
6	Occupation	
	Employed	22 (8.8)
	Unemployed	228 (91.2)
7	Planned pregnancy	
	No	41 (16.4)
	Yes	209 (83.6)
8	Total	250 (100.0)

Overall, high levels of satisfaction were reported in 232 (92.8%) women with the healthcare services that they received. The highest satisfaction rates were seen in 244 (97.6%) women for the level of medical care as well as for the amount of time spent with providers, followed by 243 (97.2%) women being satisfied with support staff care. The adequacy of health staff was also rated highly by 241 women (96.4%) (Table 2).

**Table 2:** Percentage of satisfaction/unsatisfaction for the health care domain questions

Q. No.	Health Care Domain Questions	Satisfied, N (%)	Unsatisfied, N (%)
1.	Time spent with healthcare provider	244 (97.6)	6 (2.4)
2.	Level of medical care received	244 (97.6)	6 (2.4)
3.	Care received from support staff	243 (97.2)	7 (2.8)
4.	Waiting time to be seen by healthcare provider	234 (93.6)	16 (6.4)
5.	Privacy during vaginal examinations and delivery	231 (92.4)	19 (7.6)
6.	Pain control	235 (94.0)	15 (6.0)
7.	Adequacy of health staff	241 (96.4)	9 (3.6)
8.	Overall satisfaction level		
	Low satisfaction level (≤75%)	18 (7.2)	
	High satisfaction level (>75%)	232 (92.8)	

While overall satisfaction remains high, the communication domain shows slightly lower satisfaction levels in 211 (84.4%) women compared to the general healthcare domain. The "explanation about progress serially" aspect received the lowest satisfaction rating in 219 (87.6%) women, indicating a potential area for improvement. "Healthcare provider listening to worries" received the highest satisfaction rating in 229 (91.6%) women, indicating that the healthcare providers are doing well in that area (Table 3).

**Table 3:** Percentage of satisfaction/unsatisfaction for the health worker's communication domain questions

Q. No.	Health worker's communication domain questions	Satisfied N (%)	Unsatisfied N (%)
1.	Explanation regarding condition	225 (90.0)	25 (10.0)
2.	Explanation about interventions and their need	224 (89.6)	26 (10.4)
3.	Healthcare provider listening to worries	239 (95.6)	11 (4.4)
4.	Support staff listening to worries	229 (91.6)	21 (8.4)
5.	Inclusion in formulating healthcare plan	230 (92.0)	20 (8.0)
6.	Explanation about progress serially	219 (87.6)	31 (12.4)
7.	Overall satisfaction level		
	Low satisfaction level (≤75%)	39 (15.6)	
	High satisfaction level (>75%)	211 (84.4)	

Overall, women reported high levels of satisfaction with the attitude of healthcare workers. "Courtesy and respect" from both healthcare providers and support staff received the highest satisfaction ratings in 239 (95.6%) and 236 (94.4%) women, respectively. The way staff treated family or companions, and the amount of freedom in the labor ward, both received the lowest satisfaction scores in this domain (220, 88.0%; Table 4).

**Table 4:** Percentage of satisfaction/unsatisfaction for the health worker's attitude domain questions

Q. No.	Health worker's attitude domain questions	Satisfied N (%)		Unsatisfied N (%)
1.	Courtesy, respect by healthcare provider	239 (95.6)		11 (4.4)
2.	Courtesy, respect by support staff	236 (94.4)		14 (5.6)
3.	The way staff treated you	229 (91.6)		21 (8.4)
4.	The way staff treated family or companion	220 (88.0)		30 (12.0)
5.	Amount of freedom in the labor ward	220 (88.0)		30 (12.0)
6.	Overall satisfaction level			
	Low satisfaction level (≤75%)		27 (10.8)	
	High satisfaction level (>75%)		223 (89.2)	

The environment domain showed significantly lower satisfaction levels compared to the other domains. The initiation of breastfeeding in the birthing place received the lowest satisfaction rating, with a majority of women (138, 55.2%) expressing dissatisfaction. The provision of clean toilets (78, 31.2%) and hand-washing facilities (77, 30.8%) also had high dissatisfaction ratings. Post-delivery skin-to-skin contact also had a very low satisfaction rating (Table 5).

**Table 5:** Percentage of satisfaction/unsatisfaction for the environment domain questions

Q. No.	Environment domain questions	Satisfied, N (%)	Unsatisfied, N (%)
1.	Provision of clean drinking water	207 (82.8)	43 (17.2)
2.	Provision of ventilated spaces	217 (86.8)	33 (13.2)
3.	Provision of hand washing facilities	173 (69.2)	77 (30.8)
4.	Provision of clean toilets for women	172 (68.8)	78 (31.2)
5.	Overall Facility cleanliness	204 (81.6)	46 (18.4)
6.	Allowance of post delivery Skin to skin contact	151 (60.4)	99 (39.6)
7.	Initiation of breast-feeding in birthing place	112 (44.8)	138 (55.2)
8.	Overall satisfaction level		
	Low satisfaction level (≤75%)	158 (63.2)	
	High satisfaction level (>75%)	92 (36.8)	

## Discussion

A crucial tool to reduce maternal morbidity and mortality is our knowledge about women’s satisfaction with labor and delivery care services. This knowledge, in turn, helps in increasing delivery service utilization, formulating new intervention strategies, and strengthening the existing ones [13]. The study population consisted of predominantly planned pregnancies (209, 83.6%) and 140 (56%) women who had given birth before. Even though most of the women were homemakers (228, 91.2%), it was noted that they had a wide range of educational backgrounds, with 169 (67.6%) women from urban areas, and were young (mean age 27.04±4.46 years). Although the questionnaire tool was administered in both Hindi and English, most of the women used the Hindi version (242, 96.0%). This highlights the importance of Hindi language services in the hospital. Similar population demographics were observed by Deepak Shrestha et al., showing that most women were under 35 years and educated homemakers [14].

In this study, 232 (92.8%) women were completely satisfied in the entire health care domain, whereas in the health workers' communication domain, only two items fell short, that is, explanation about interventions and serial progress of labor. In the domain of attitude of healthcare workers, there was a slight dissatisfaction in the way that the family/birth companions were treated, as well as the freedom of mobility in labor wards. The environment domain showed significantly lower satisfaction levels compared to the other domains, especially the initiation of breastfeeding in the birthing place. It is well understood that satisfied women are more likely to return in the future and recommend the institution to their relatives/friends [15,16].

In the domain dealing with overall health care, a very small number of women showed low overall satisfaction (18, 7.2%). The two items on the questionnaire tool that got the highest satisfaction responses were the time spent with healthcare providers and the level of healthcare received. However, issues related to waiting time and privacy during examinations and delivery were identified as areas where improvements could be made. Similar to our study, the Adekanye et al. study revealed that approximately half of the patients experienced delays, often due to extended waiting times in accessing care [17].

Our study brought out the humane aspect of healthcare communication, wherein 229 (91.6%) women reported that their worries were being well heard by doctors and support staff as well. The “birth in Brazil” national research study showed findings that resonate well with our study with respect to clarity of the information provided, which was lacking behind in other items in this communication domain [8]. Studies demonstrate the women's perception that they are active participants during childbirth and are well informed about the progress of labor is associated with high levels of positive care assessment [18,19]. Hence, to avoid passive childbirth care practices, this area needs to be addressed.

For the healthcare worker’s attitude domain, the overall satisfaction level was high, but 27 (10.8%) women with low overall satisfaction warranted remedial steps, as seen in our study. Despite other attitude items being highly rated, it was noted that the freedom of mobility in labor wards and attitude towards family/companion were rated low; this was similar to studies done in Nepal [14] and Ethiopia [20], as well as in a Cochrane review [21]. This lacuna eventually can hamper women’s compliance and overall childbirth experience.

As per our study findings, the lowest satisfaction scoring domain was seen in the environment domain. The major concerns were breastfeeding and clean provisions. Other studies that had similar concerns showed that 49.8% of mothers expressed their dissatisfaction with the support received for breastfeeding [20] and in the Indian context also, 31% of the participants indicated satisfaction with the cleanliness of both the toilet and hospital ward [22]. In 2020, WHO designed a tool for health personnel to meet this need. The tool is known as the labor care guide (LCG) and it aims to stimulate shared decision-making by health-care providers and women and promote women-centered care. Thus, proper incorporation of the WHO LCG tool can effectively lead to higher maternal satisfaction and a positive outcome for both mother and baby [23]. Our research faced certain constraints, including a limited sample size and being conducted at a single facility. These restrictions could be overcome in future research by adopting a multi-center study design.

## Conclusions

There is a palpable need for asserting satisfactory behavior towards the family and woman as a whole. Despite the medical standards of care being met well, the communication and healthcare worker's attitudes can be improved. Healthcare alone cannot be the only pillar of maternal satisfaction and it is a mixed outcome of healthcare as well as resources, as is evident low overall scores in the environmental domain. There has to be a shift from passive childbirth care being currently practiced in tertiary care centers towards active participation from the woman for a more positive childbirth experience.

## Figures and Tables

**Figure 1:**
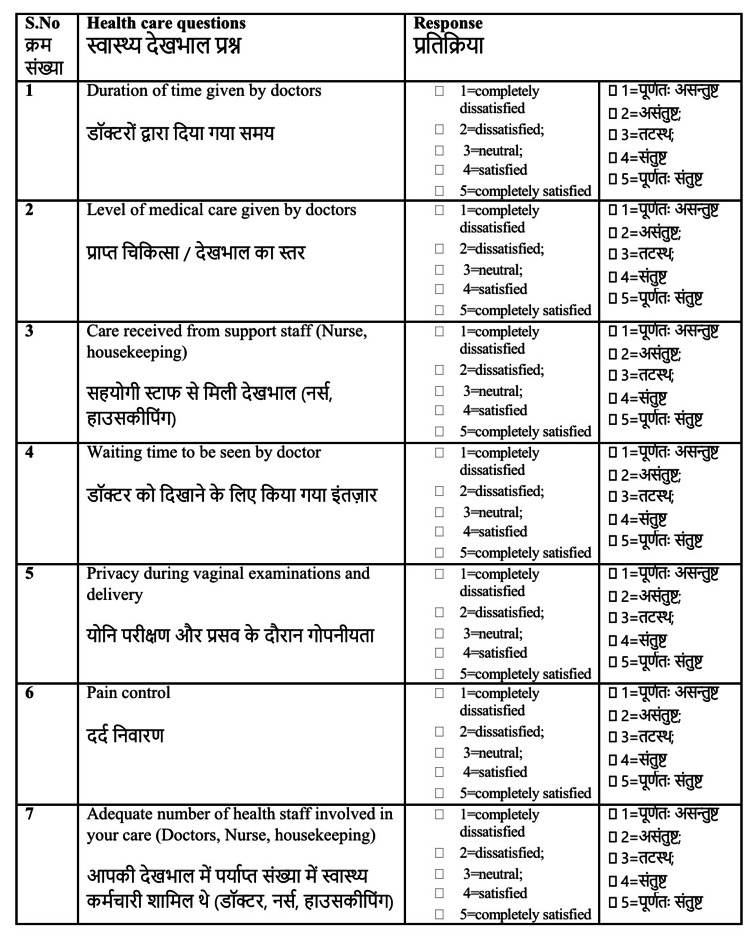
Health care domain questions

**Figure 2:**
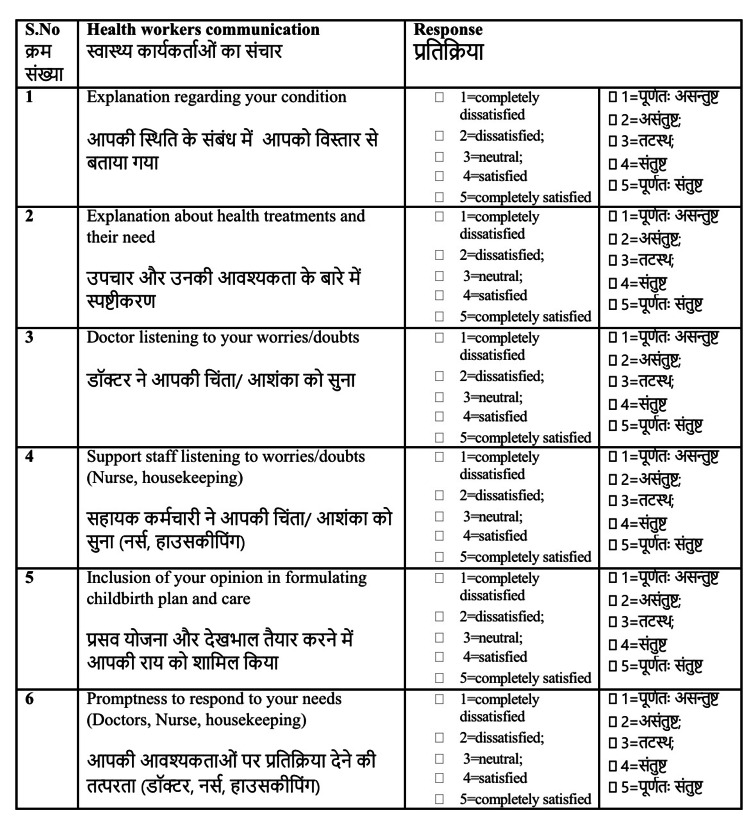
Health worker's communication domain questions

**Figure 3:**
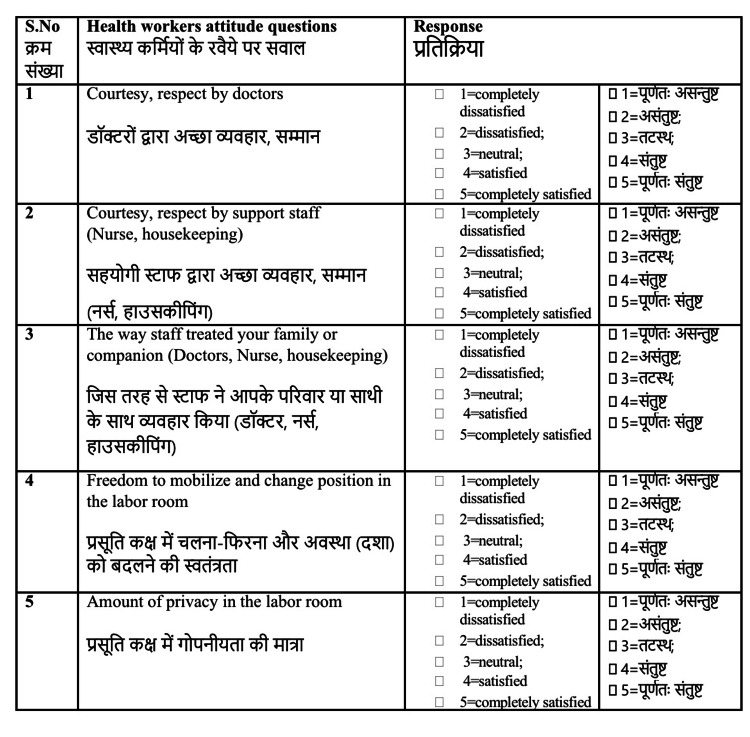
Health worker's attitude domain questions

**Figure 4:**
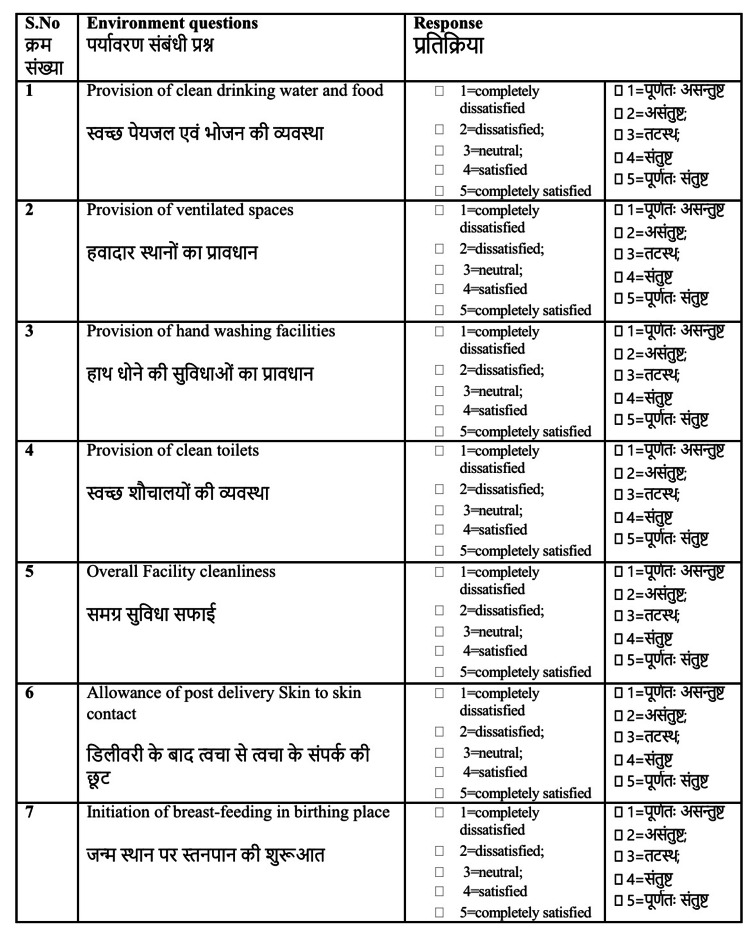
Environment domain questions

## Appendices

Study Questionnaire (शोध प्रश्नावली)

Title of study: Assessment of Women’s Satisfaction of Childbirth Experience in a Tertiary Care Hospital in Central India

शोध का शीर्षक: मध्य भारत के एक मेडिकल कॉलेज अस्पताल में प्रसव के अनुभव से महिलाओं की संतुष्टि का आकलन

On a scale of 1 to 5 please respond to the below mentioned questions as per your perceived level of satisfaction.

1=completely dissatisfied;2=dissatisfied; 3=neutral;4=satisfied;5=completely satisfied

1 से 5 के पैमाने पर कृपया अपनी संतुष्टि के अनुमानित स्तर के अनुसार नीचे दिए गए प्रश्नों का उत्तर दें।

1=पूर्णतः असंतुष्ट;2=असंतुष्ट; 3=तटस्थ;4=संतुष्ट;5=पूर्णतः संतुष्ट

Name(नाम):

Age (आयु):

Education (शिक्षा का स्तर):                 

Contact No (फ़ोन नंबर):                                        

Address (पता):                                               

Occupation (व्यवसाय):

Obstetric History

GPLA

Language of preference (भाषा):  English/ हिंदी

IPD number :

Planned pregnancy (वांछित गर्भावस्था) :     Yes/No (हाँ / नहीं)

The questionnaire consists of 25 items/variables grouped into four domains - health care (seven questions) (Figure 1); health worker's communication (six questions) (Figure 2); health worker's attitude (five questions) (Figure 3); environment (seven questions) (Figure 4).

Health care domain (seven questions) (Figure 1).

Health worker's communication domain (six questions) (Figure 2).

Health worker's attitude domain (five questions) (Figure 3).

Environment domain (seven questions) (Figure 4).
